# A comparison between two different definitions of contrast-associated acute kidney injury for long-term mortality in patients with diabetes undergoing coronary angiography: a prospective cohort study

**DOI:** 10.1186/s12872-020-01778-6

**Published:** 2020-11-16

**Authors:** Zhubin Lun, Li Lei, Dianhua Zhou, Ming Ying, Liwei Liu, Guanzhong Chen, Jin Liu, Yibo He, Huanqiang Li, Zhidong Huang, Yongquan Yang, Jianfeng Ye, Yong Liu

**Affiliations:** 1 The First School of Clinical Medicine, Guangdong Medical University, Zhanjiang, 523808 China; 2 Department of Cardiology, Dongguan TCM Hospital, Dongguan, 523000 China; 3 Department of Cardiology, Guangdong Provincial Key Laboratory of Coronary Heart Disease Prevention, Guangdong Cardiovascular Institute, Guangdong Provincial People’s Hospital, The Affiliated Guangdong Provincial People’s Hospital of South China University of Technology, Guangdong Academy of Medical Sciences, Guangzhou, 510080 China; 4 The Second School of Clinical Medicine, Southern Medical University, Guangzhou, 510515 China

**Keywords:** Contrast-associated acute kidney injury, Diabetes, Definitions, Long-term mortality, Population attributable risks

## Abstract

**Background:**

The definitions of contrast-associated acute kidney injury (CA-AKI) are diverse and have different predictive effects for prognosis, which are adverse for clinical practice. Few articles have discussed the relationship between these definitions and long-term prognosis in patients with diabetes.

**Methods:**

A total of 1154 diabetic patients who were undergoing coronary angiography (CAG) were included in this study. Two definitions of CA-AKI were used: CA-AKI_A_ was defined as an increase ≥ 0.3 mg/dl or > 50% in serum creatinine (SCr) from baseline within 72 h after CAG, and CA-AKI_B_ was defined as an increase ≥ 0.5 mg/dl or > 25% in SCr from baseline within 72 h after CAG. We used Cox regression to evaluate the association of these two CA-AKI definitions with long-term mortality and calculate the population attributable risks (PARs) of different definitions for long-term prognosis.

**Results:**

During the median follow-up period of 7.4 (6.2–8.2) years, the overall long-term mortality was 18.84%, and the long-term mortality in patients with CA-AKI according to both CA-AKI_A_ and CA-AKI_B_ criteria were 36.73% and 28.86%, respectively. We found that CA-AKI_A_ (HR: 2.349, 95% CI 1.570–3.517, *p* = 0.001) and CA-AKI_B_ (HR: 1.608, 95% CI 1.106–2.339, *p* = 0.013) were associated with long-term mortality. The PARs were the highest for CA-AKI_A_ (31.14%), followed by CA-AKI_B_ (14.93%).

**Conclusions:**

CA-AKI is a common complication in diabetic patients receiving CAG. The two CA-AKI definitions are significantly associated with a poor long-term prognosis, and CA-AKI_A_, with the highest PAR, needs more clinical attention.

## Introduction

Contrast-associated acute kidney injury (CA-AKI) is a common complication after coronary angiography that is closely related to adverse clinical prognoses such as cardiovascular disease, renal events, and long-term mortality [1–3]. CA-AKI is usually defined as an increase in serum creatinine (SCr) ≥ 50% or ≥ 0.3 mg/dl within 72 h after exposure to a contrast agent, but the definition of CA-AKI is not limited to this. It is defined as an increase in SCr by 0.5 mg/dl or 25% within 72 h after using a contrast agent, which has also been confirmed in many clinical studies. In fact, the incidence and prognostic value of CA-AKI are different with different definitions [4–8]. It has been found that different definitions have different efficacies in predicting long-term mortality in STEMI patients, and the criteria of the Acute Kidney Injury Network (AKIN) has better efficacy [4]. Different definitions also have different performances in terms of population-attributable risk (PAR) in AMI patients; the standard definition, which is an increase in SCr ≥ 50% or ≥ 0.3 mg/dl within 72, has the highest PAR [6]. In previous studies, diabetes mellitus was considered a risk factor for CA-AKI, and it has been applied to several high-efficacy predictive models for CA-AKI [9–11]. However, to date, there are few articles comparing the association between different definitions and long-term prognosis in diabetic patients.

Therefore, we evaluated the association between different definitions of CA-AKI and long-term mortality in diabetic patients after coronary angiography in this article.

## Methods

### Study design and population

This is a single-center prospective observational study (PROCOMIN, ClinicalTrials.gov NCT01400295). From January 2010 to December 2013, 1154 consecutive patients aged ≥ 18 years who were diagnosed with diabetes underwent coronary angiography in Guangdong Provincial People's Hospital and were included in the study. Exclusion criteria included pregnancy, lactation, intravascular administration of contrast media within 7 days before the operation or 3 days after the operation, no use of low osmotic pressure contrast media, cardiovascular surgery or intravascular repair, end-stage renal disease or kidney replacement, creatinine deficiency before or after the operation, malignant tumor and no use of isotonic saline for hydration [12]. The research was approved by the ethics research committee of our institute, and patient informed consent was obtained (approval number: GDREC2010112H).

### Study protocol

According to the current guidelines, patients underwent coronary angiography, including standard clinical practice with standard guide catheters/guidewires/balloon catheters/stents via the femoral or radial approach. Contrast media, drugs and intra-aortic balloon pump (IABP) support were all used by cardiovascular physicians according to the patients' conditions and guidelines. In addition, all patients were administered nonionic hypotonic early contrast media. From 2–12 h before surgery to 6–24 h after surgery, isotonic saline was given at a rate of 1 ml/kg/h, and the hydration rate of patients with heart failure and a left ventricular ejection fraction ≤ 40% was halved. SCr concentrations were measured on days 1, 2, and 3 in all patients before and after surgery.

### Clinical definitions and follow-up

Long-term mortality was defined as all-cause death at the latest follow-up. Diabetes was defined as a previous diagnosis of diabetes or an HbAlc level ≥ 6.5 (48 mmol/mol). CA-AKI_A_ was defined as an increase in serum creatine by 0.3 mg/dl or 50% within 72 h after the procedure, while CA-AKI_B_ was defined as an increase in SCr by 0.5 mg/dl or 25% within 72 h after the procedure [13–16]. Anemia was defined as a baseline hematocrit value < 39% for men or < 36% for women according to the World Health Organization criteria [17]. Congestive heart failure (CHF) was defined as New York Heart Association (NYHA) functional class > 2, Killip class > 1 or pulmonary edema. SBP < 80 mmHg for at least 1 h during the perioperative use of positive inotropic force or hemodynamic balance with IABP was considered hypotension. The formula for eGFR used was the modified diet in renal disease formula (186 × SCr (mg/dL) − 1.154 × age − 0.203 × (0.742 for women). At 3, 6, 12, 18, and 24 months after coronary angiography, office follow-up visits and telephone interviews were conducted by professionals, and subsequent clinical events were monitored and recorded.

### Statistical analysis

Continuous variables are presented as the mean ± SD or median ± IQR, and we used Wilcoxon rank-sum tests or Student’s t-test to compare the differences between two independent samples (with CA-AKI and without CA-AKI) using different definitions. For categorical variables expressed as counts (percentages), we used the Chi-square test or Fisher’s exact test to verify the difference. For the first step, we used a univariate logistic model to analyze the data to identify the predictors of CA-AKI_A_ and CA-AKI_B_. The results of this analysis are shown as odds ratios and 95% confidence intervals (CIs). Multivariate logistic analysis was also used. Kaplan–Meier analysis was used to calculate cumulative mortality using different definitions, and the differences between the curves were evaluated with the log-rank test. We used a multivariate Cox regression model adjusting for other risk factors (e.g., age, AMI, hypotension, HS-CRP) to show the relationship between CA-AKI and long-term mortality. The adjusted risk factors in the univariate Cox regression analysis were selected based on previous studies and clinical importance [18, 19]. Two multivariate Cox proportional hazard regression models were applied for the two different definitions of CA-AKI. The PAR was calculated by the following equation: PAR = P(HR − 1)/[1 + P(HR − 1)], where p is the incidence of CA-AKI using different definitions in the database. The standard error of the PAR was calculated using the delta method. A two-sided probability value < 0.05 was considered significant. All data analyses were conducted with SAS version 9.4 (SAS Institute, Cary, NC) and R software (version 4.0.0; R Core Team, Vienna, Austria).

## Results

### Patient characteristics

A total of 1154 consecutive diabetic patients who received coronary angiography were included in the analysis. The demographic, clinical and procedural characteristics of patients with or without CA-AKI are shown in Table 1. Overall, the mean age was 64.46 ± 10.55 years, with females accounting for just under one-third of the population (27.73%). Only 213 patients (18.5) were age ≥ 75 years. The number of persons diagnosed with CAD, CHF and eGFR < 60 ml/min/1.73 m^2^ was 1074, 733 and 268, respectively. The usage rates of ACEIs/ARBs, beta-blockers, statins and diuretics were 89.08%, 84.84%, 97.57% and 22.62%, respectively. A total of 710 (61.53%) patients underwent PCI. The mean dose of contrast agent was 131.55 ± 63.85, and the median follow-up period of 7.4 (6.2–8.2) years.

**Table 1:** Baseline characteristics

Variables	Total (n = 1154)	CA-AKI_A_			CA-AKI_B_		
		Yes (n = 98)	No (n = 1056)	*p* value	Yes (n = 149)	No (n = 1005)	*p* value
Age, y	64.46 ± 10.55	70.24 ± 9.45	63.92 ± 10.49	< 0.001	66.42 ± 11.41	64.17 ± 10.39	0.024
Age ≥ 75, n (%)	213 (18.5)	37 (37.8)	176 (16.7)	< 0.001	41 (27.5)	172 (17.1)	0.003
Female sex, n (%)	320 (27.73)	34 (34.69)	286 (27.08)	0.107	49 (32.89)	271 (26.97)	0.132
Weight, kg	66.48 ± 10.68	64.3 ± 11.42	66.68 ± 10.59	0.050	65.60 ± 11.45	66.61 ± 10.56	0.312
SBP, mmHg	131.12 ± 20.80	131.42 ± 27.21	131.09 ± 20.12	0.906	129.00 ± 24.58	131.43 ± 20.17	0.251
DBP, mmHg	76.65 ± 12.06	77.10 ± 13.99	76.61 ± 11.87	0.738	76.64 ± 13.41	76.65 ± 11.85	0.988
HR, bpm	76.39 ± 13.99	81.13 ± 17.17	75.95 ± 13.59	0.005	79.01 ± 16.14	76.00 ± 13.61	0.032
CHF, n (%)	733 (63.52)	79 (80.61)	654 (61.93)	< 0.001	106 (71.14)	627 (62.39)	0.042
CAD, n (%)	1074 (93.07)	96 (97.96)	978 (92.61)	0.054	139 (93.29)	935 (93.03)	0.991
Hypotension, n (%)	22 (1.91)	9 (9.18)	13 (1.23)	< 0.001	11 (7.38)	11 (1.09)	< 0.001
LVEF, %	56.97 ± 13.05	50.54 ± 13.57	57.59 ± 12.84	< 0.001	53.15 ± 13.72	57.56 ± 12.85	< 0.001
LVEF < 40%, n (%)	119 (10.31)	18 (18.37)	101 (9.56)	0.011	23 (15.44)	96 (9.55)	0.046
Hypertension, n (%)	777 (67.33)	75 (76.53)	702 (66.48)	0.044	103 (69.13)	674 (67.06)	0.628
Anemia, n (%)	407 (35.27)	49 (50.00)	358 (33.90)	0.001	56 (37.58)	351 (34.93)	0.561
AMI, n (%)	379 (32.84)	56 (57.14)	323 (30.59)	< 0.001	76 (51.01)	303 (30.15)	< 0.001
eGFR < 60 ml/min/1.73 m^2^, n (%)	268 (23.2)	33 (33.7)	235 (22.3)	0.016	41 (27.5)	227 (22.6)	0.230
LDL-C, mmol/L	2.67 ± 0.96	2.99 ± 1.05	2.65 ± 0.95	0.009	2.97 ± 1.28	2.63 ± 0.90	0.007
HDL-C, mmol/L	1.01 ± 2.20	0.94 ± 0.24	1.02 ± 2.28	0.340	0.95 ± 0.26	1.02 ± 2.35	0.365
HS-CRP, mg/L	19.02 ± 37.45	38.72 ± 51.47	17.04 ± 35.18	< 0.001	31.99 ± 46.06	16.91 ± 35.45	< 0.001
SCR, μmol/L	96.09 ± 47.36	126.4 ± 62.6	93.28 ± 44.69	< 0.001	96.07 ± 54.58	96.10 ± 46.22	0.996
Hemoglobin, g/L	131.03 ± 16.30	122.34 ± 20.22	131.74 ± 15.73	< 0.001	130.11 ± 18.34	131.15 ± 16.01	0.552
HbA1c, %	7.60 ± 1.53	7.79 ± 1.60	7.58 ± 1.52	0.250	130.11 ± 18.34	131.15 ± 16.01	0.830
Serum albumin, g/L	35.09 ± 4.59	31.81 ± 5.27	35.35 ± 4.43	< 0.001	33.90 ± 5.58	35.25 ± 4.43	0.013
ACEI/ARB, n (%)	1028 (89.08)	81 (82.65)	947 (89.68)	0.033	131 (87.92)	897 (89.25)	0.626
Beta-blocker, n (%)	979 (84.84)	71 (72.45)	908 (85.98)	< 0.001	120 (80.54)	859 (85.47)	0.110
Statin, n (%)	1126 (97.57)	94 (95.92)	1032 (97.73)	0.290	145 (97.32)	981 (97.61)	0.776
Diuretics, n (%)	261 (22.62)	49 (50.00)	212 (20.08)	< 0.001	57 (38.26)	204 (20.30)	< 0.001
Metformin, n (%)	79 (6.85)	2 (2.04)	77 (7.29)	0.085	7 (4.70)	72 (7.16)	0.422
PCI, n (%)	710 (61.53)	58 (59.18)	652 (61.74)	0.160	89 (59.73)	621 (61.79)	0.174
CV, mL	131.55 ± 63.85	137.09 ± 63.05	131.04 ± 63.93	0.366	130.27 ± 63.2	131.74 ± 63.98	0.791
Periprocedure IABP, n (%)	59 (5.11)	25 (25.51)	34 (3.22)	< 0.001	23 (15.44)	36 (3.58)	< 0.001
Mehran score	6.37 ± 4.53	11.08 ± 6.35	5.92 ± 4.04	< 0.001	8.36 ± 6.09	6.07 ± 4.17	< 0.001

*CA-AKI* contrast-associated acute kidney injury, *SBP* systolic blood pressure, *DBP* diastolic blood pressure, *HR* heart rate, *CHF* chronic heart failure, *CAD* coronary artery disease, *LVEF* left ventricular ejection fraction, *LDL-C* low-density lipoprotein-C, *HDL-C* high-density lipoprotein-C, *HS-CRP* high-sensitivity C-reactive protein, *SCR* serum creatinine, *Lpa* lipoprotein a, *eGFR* estimated glomerular filtration rate, *ACEI* angiotensin-converting enzyme inhibitor, *ARB* angiotensin receptor blocker, *PCI* percutaneous coronary intervention, *CV* contrast volume, *IABP* intra-aortic balloon pump

### Incidence of CA-AKI by the CA-AKI_A_ and CA-AKI_B_ criteria

According to the CA-AKI_A_ criteria, CA-AKI occurred in 98 patients (8.49%), while according to the CA-AKI_B_, CA-AKI occurred in 149 patients (12.91%). Among 98 patients with CA-AKI_A_, 86 patients met the criteria for CA-AKI_B_. On the other hand, among 149 patients with CA-AKI_B_, 86 patients met the criteria for CA-AKI_A_. However, regardless of the definition of CA-AKI, patients with CA-AKI were older, had a reduced LVEF, and had more basic diseases. The usage of ACEIs/ARBs and beta-blockers in patients with CA-AKI_A_ was lower, but there was no significant difference from those with CA-AKI_B_. Similarly, we found no significant association between CA-AKI and non-CA-AKI patients in terms of statin drug use (Table 1).

### Incidence and predictors of long-term mortality

**Fig. 1:**
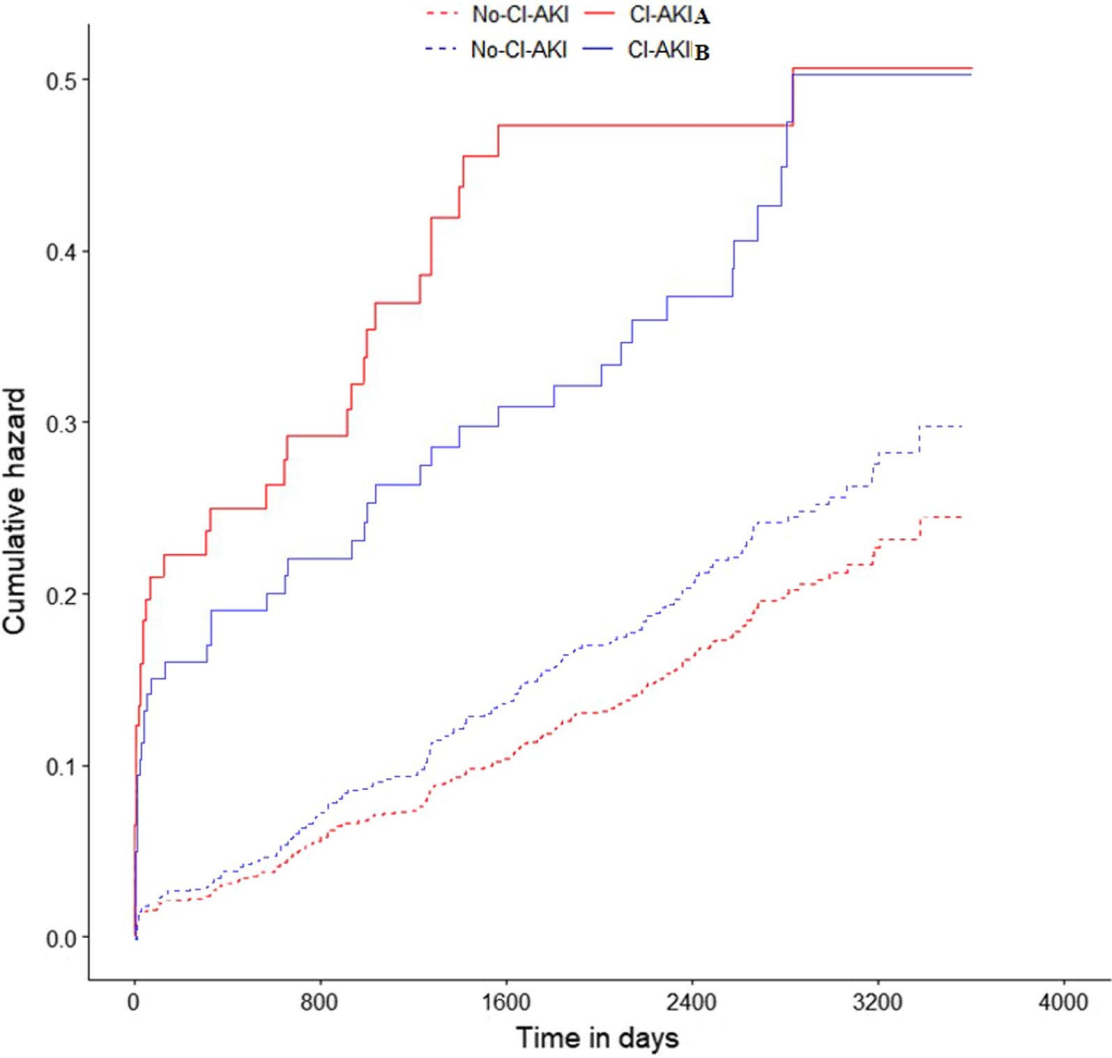
Association between contrast-associated acute kidney injury and long-term mortality in diabetic patients with undergoing coronary angiography

During the median follow-up period of 7.4 (6.2–8.2) years, the overall long-term mortality was 18.84%, and the long-term mortality in patients with CA-AKI according to the CA-AKI_A_ and CA-AKI_B_ criteria was 36.73% and 28.86%, respectively. Kaplan–Meier curves showed that the long-term prognosis of patients with CA-AKI was worse than that of those without CA-AKI according to the two definitions of CA-AKI (log-rank *p* < 0.01; Fig. 1).

**Fig. 2:**
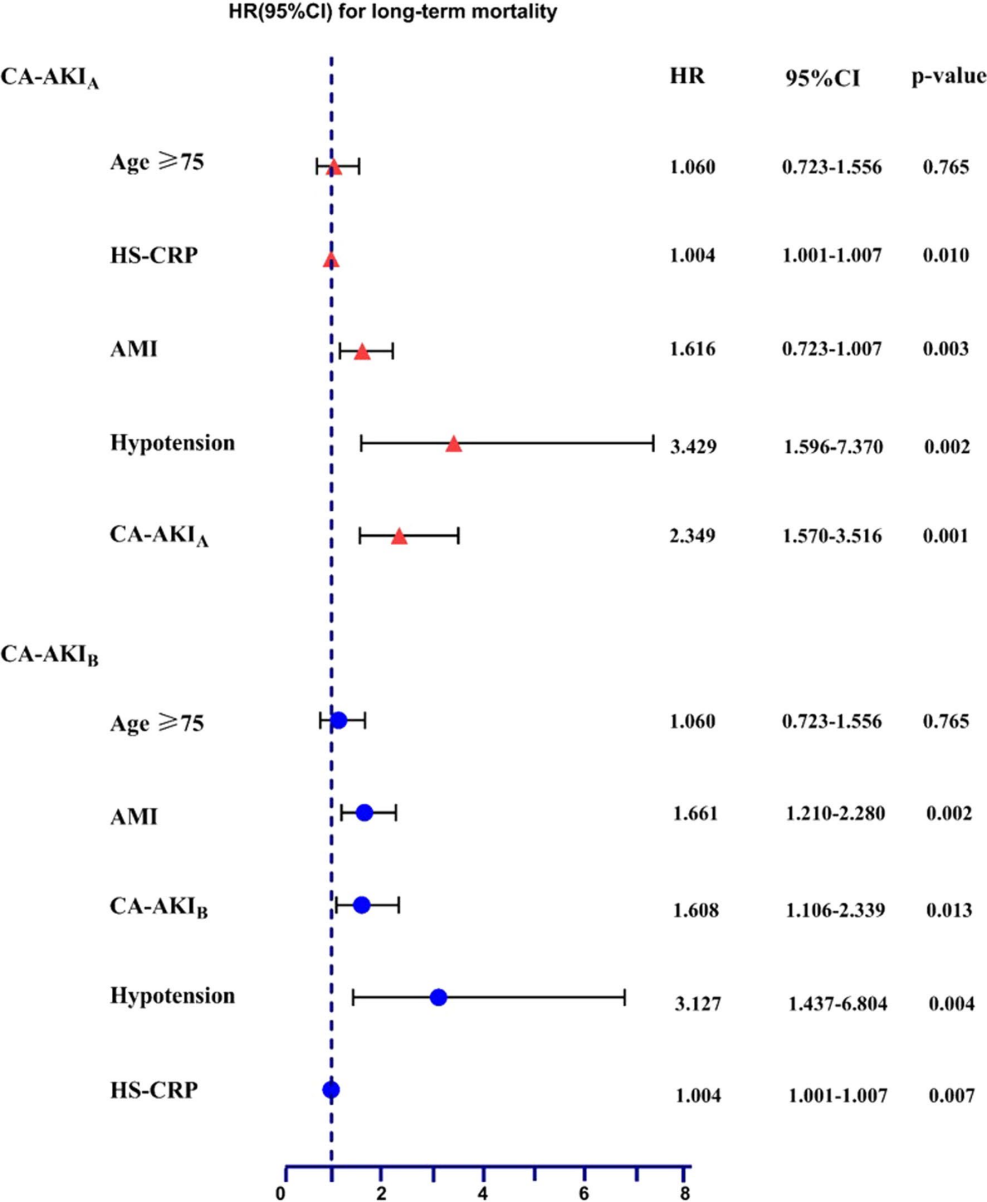
Multivariable analysis of risk factors for long-term mortality

The univariate and multivariate analyses of risk factors for long-term mortality are shown in Table 2 and Fig. 2. The analysis showed that the independent predictive factors related to long-term mortality are age ≥ 75 years, AMI, hypotension, HS-CRP and CA-AKI. As expected, there was an increased risk of long-term mortality associated with CA-AKI. CA-AKI_A_ and CA-AKI_B_ increased the long-term mortality risk by 2.349 and 1.608 times, respectively (95% CI 1.570–3.516 and 95% CI 1.106–2.339, respectively).

**Table 2:** Univariable analysis of risk factors for long-term mortality

	Univariable analysis		
	HR	95% CI	*p* value
Age ≥ 75	1.016	1.002–1.029	0.021
Female sex	0.976	0.723–1.318	0.875
Weight	0.995	0.983–1.008	0.472
Smoking	1.065	0.846–1.341	0.593
CHF	1.545	1.134–2.106	0.006
AMI	1.837	1.404–2.402	< 0.001
Anemia	1.192	0.906–1.569	0.211
eGFR < 60 ml/min/1.73 m^2^	1.127	0.828–1.534	0.448
Hypertension	0.912	0.690–1.205	0.517
Hypotension	4.047	2.149–7.661	< 0.001
HS-CRP, mg/L	1.006	1.003–1.009	< 0.001
ACEI/ARB	0.702	0.480–1.026	0.068
Beta-blocker	0.649	0.468–0.902	0.010
Diuretic	1.486	1.175–1.881	0.001
IABP	3.883	1.443–10.450	0.007
CV	1.001	0.999–1.003	0.415
CA-AKI_A_	2.821	1.973–4.038	< 0.001
CA-AKI_B_	1.926	1.380–2.690	< 0.001

*CA-AKI* contrast-associated acute kidney injury, *CHF* chronic heart failure, *AMI* acute myocardial infarction, *HS-CRP* high-sensitivity C-reactive protein, *ACEI* angiotensin-converting enzyme inhibitor, *ARB* angiotensin receptor blocker, *PCI* percutaneous coronary intervention, *CV* contrast volume, *IABP* intra-aortic balloon pump

### PARs of CA-AKI by CA-AKI_A_ and CA-AKI_B_ criteria

**Fig. 3:**
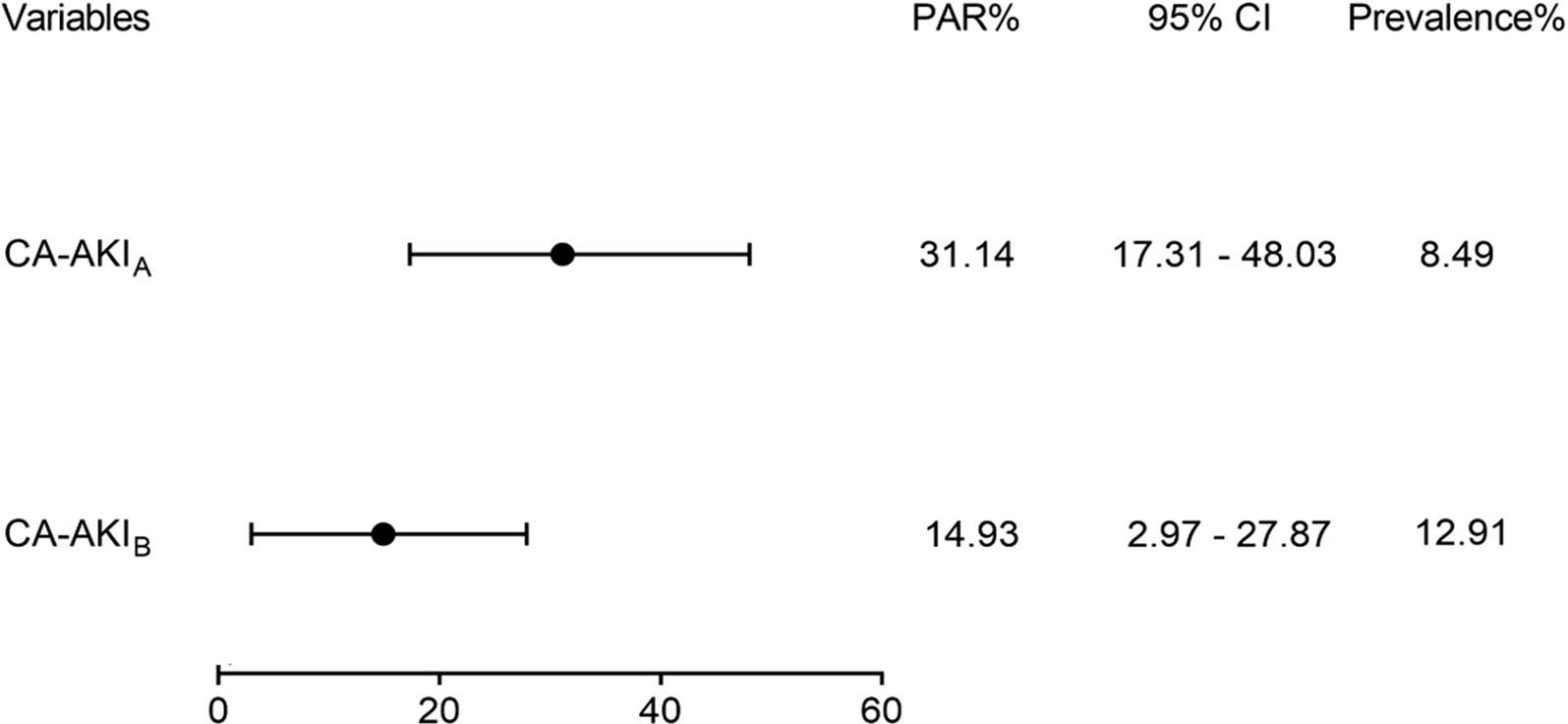
Population attributable risk of two different definitions of contrast-associated acute kidney injury

Between the two definitions of CA-AKI, the prevalence of CA-AKI_A_ was 8.49%, while the prevalence of CA-AKI_B_ was 12.91%. The PAR of CA-AKI_A_ was significantly higher than that of CA-AKI_B_ [31.14% (95% CI 17.31–48.03%) vs 14.93% (95% CI 2.97–27.87%)] (Fig. 3).

## Discussion

We analyzed the relationship between different definitions of CA-AKI and long-term prognosis in diabetic patients and compared the PARs of long-term mortality. According to our findings, the incidence of CA-AKI_A_ was slightly lower than that of CA-AKI_B_, while the mortality rate was the opposite. In addition, we also found that the mortality rate of patients with CA-AKI was higher than that of patients without CA-AKI. After adjusting for demography, drugs and cardiorenal risk factors, an increased risk of long-term mortality was associated with CA-AKI_A_ and CA-AKI_B_. However, CA-AKI_A_ was associated with higher risk of long-term mortality and PAR than CA-AKI_B_.

Without a uniform definition, the incidence of CA-AKI was between 8.49 and 12.91% in patients with diabetes mellitus undergoing coronary angiography. The incidence of CA-AKI in patients with diabetes indicated in Chalikias's review was similar [20]. Our results suggest that regardless of how CA-AKI is defined, it leads to a poor long-term prognosis. In a study discussing the clinical significance of CA-AKI, CA-AKI was associated with death compared with non-CA-AKI, even when different definitions of CA-AKI were applied [5]. This further confirmed those results. On the other hand, in a study with a median follow-up period of 365 ± 130 days, Centola et al. compared the effects of different definitions of CA-AKI on long-term death in 402 patients with STEMI and found that each different definition increased the risk of death [4]. CA-AKI may be an important risk factor for long-term mortality, rather than an intermediate, which is similar to Mehran's recently described viewpoint [21]. The research of Lassnigg et al. also supports this view, and they found that a small increase or decrease in serum creatinine was significantly related to death [22]. Fluctuation in serum creatinine may be due to unstable hemodynamics, and the downstream adverse events have little relevance.

As far as we know, few studies have compared the relationship between different definitions of CA-AKI and long-term mortality in diabetic patients. However, we evaluated the correlation between the two definitions of CA-AKI and long-term mortality in diabetic patients by cox regression analysis and PAR calculation. The significance of population attributable risk (PAR) is the proportion of cases that would not happen in the population without risk factors. Chen et al. also found that the 1^st^ most common definition of CA-AKI (≥ 0.3 mg/dl or ≥ 50%) and the 2^nd^ most common definition of CA-AKI (≥ 0.5 mg/dl or ≥ 25%) have different predictive powers for long-term mortality risk, and the 1st most common definition of CA-AKI is more closely related to all-cause death [5]. Recently, Lei et al. found that three different definitions of CA-AKI were also inconsistent in predicting death in AMI patients. Their conclusion was that the highest PAR was found in CA-AKI_A_ (a SCr elevation ≥ 50% or ≥ 0.3 mg/dl within 72 h), which is consistent with our results [6]. Benoit Guillon and his colleagues showed that the CA-AKI defined by SCr absolute increase ≥ 0.3 mg/dl or ≥ 50% over baseline was strongly correlated with 6 months all-cause death in patients with CAG [7]. And Gabriele Pesarini et al. found that an absolute increase of SCr > 0.3 mg/dl seems to be the most clinically meaningful cut-off value for CA-AKI and continuous renal damage monitoring [23]. These studies have shown that different definitions of CA-AKI have different strengths of association with death, and CA-AKI defined as SCr elevation ≥ 50% or ≥ 0.3 mg/dl within 72 h has a stronger association with long-term mortality. This further confirmed our results. Our results show that the HR and PAR of CA-AKI_A_ were higher than those of CA-AKI_B_, which indicates that the CA-AKI_A_ definition is more closely related to long-term mortality. On the one hand, this may be related to the patient we included, and diabetic patients belong to high-risk groups. In a long-term hyperglycemic environment, HbAlc increases, oxygenated hemoglobin decreases, and microvascular perfusion is insufficient, which leads to kidney ischemia and hypoxia and aggravated kidney injury. On the other hand, when the "restrictive" criteria are adopted (an increase ≥ 0.5 mg/dl in SCr above baseline), patients with slight increase in serum creatinine may be ignored, which will have an impact on long-term death. When the "relativity" criteria (an increase in SCr > 25% above baseline) are adopted, the recognition ability will be reduced [24]. For example, in Gajewska's research, it was found that the renal damage caused by SCr increased from 1.2 to 1.6 mg/dl was much higher than that caused by SCr increased from 3.0 to 3.75 mg/dl [25]. In addition, in previous studies, mild increases in serum creatinine in patients with lower baseline serum creatinine are more likely to lead to significant deterioration of renal function. The majority of diabetic patients often have renal insufficiency, and a slight increase in "restrictive" criteria (≥ 0.3 mg/dl or ≥ 0.5 mg/dl) often cannot truly reflect the occurrence of kidney injury in diabetic patients. The "relativity" criteria are often more able to reflect the exact kidney damage based on the poor baseline renal function.

After adjusting for AMI, hypotension and HS-CRP, we found that CA-AKI was significantly associated with long-term mortality. AMI, hypotension and HS-CRP are the research hotspots of CA-AKI and prognosis. The association between AMI and CA-AKI has been confirmed in previous studies [26, 27], and Zafrir et al. also found that patients with AMI can have significantly increased long-term mortality [28]. Hypotension, as a strong predictor of CA-AKI, is also a good predictor of death. Compared with the risk found in our long-term follow-up, Chong et al. found that patients with hypotension had a high risk of death at 6 months [29]. As for HS-CRP, it is an important indicator of inflammation. In Xiao-Sheng Guo's et al. study, the inflammatory response was found to be an important mechanism for the occurrence of CA-AKI, and the risk of long-term mortality was also increased with an HS-CRP level > 7.3 mg/l [30]. The reliability of our analysis can be further enhanced by adjusting for variables of clinical significance.

At present, there were various definitions of CA-AKI [20], which makes clinicians confused about the diagnosis of CA-AKI. We confirmed that CA-AKI is an independent risk factor for long-term mortality in patients with diabetes, and the definition of CA-AKI_A_ is more suitable for diabetic patients than CA-AKI_B_ for assessing the relationship between CA-AKI and prognosis. At the same time, the strict definition of CA-AKI can prompt a poor prognosis. This is helpful for clinicians to identify and manage high-risk CA-AKI patients that are highly related to long-term death, which improving the prognosis. We found that among patients with diabetes, the definition of CA-AKI_A_ is more suitable for assessing the relationship between disease and prognosis, and more attention and care should be given to CA-AKI_A_ patients in clinical practice. The comparison of the definition is helpful for medical staff to identify the patients with CA-AKI who are most related to long-term adverse prognosis in high-risk groups (such as heart failure, chronic kidney disease, anemia, or diabetes), so as to carry out accurate management. This method of using cox regression analysis and PAR for comparison of definitions in high-risk patients to guide clinical management is worthy of promotion. Our study did not evaluate the impact of the progression of renal function on long-term mortality due to the lack of SCr during follow-up. In the study of Nemoto et al., patients with CA-AKI were more likely to suffer persistent impairment of renal function, which strongly affects long-term prognosis [31]. This may affect our results and has become a major limitation of our research.

Our research has several limitations. First, the patients we included in the study were diabetic patients, which will reduce the generalizability of the findings. Second, our study was only a sub-study of a single-center prospective observational study in South China, and the prevalence of CA-AKI was not significant. However, we are the first to explore the relationship between the definition of CA-AKI and long-term prognosis in diabetic patients, and we also found that our results were similar to those reported in previous studies involving high-risk groups. Third, we conclude that preventing the occurrence of CA-AKI can reduce the risk of long-term mortality but cannot directly confirm this conclusion. However, our study evaluated the association between CA-AKI and long-term death by calculating PAR and further verified the results of the multivariate analysis. Fourth, due to the limitation of the sample size of our study, the variables for multivariable analysis adjustment were limited. However, long-term mortality (all-cause mortality) was multifactorial and complicated. In addition, most of the patients included in our study had coronary artery disease. During follow-up, they may be more prone to cardiovascular mortality. This long-term mortality (all-cause mortality) is more close to cardiovascular mortality. Fifth, we lacked the SCr data of patients during the follow-up and were unable to assess the progression of renal insufficiency, which may have an impact on our results. However, we also adjusted for clinically significant variables related to long-term mortality in the multivariate analysis, which makes our results more credible. Future studies still need to supplement the assessment of patients' renal function and the endpoint of cardiovascular mortality to improve the credibility of the results.

## Conclusion

Our conclusion proves that CA-AKI is an independent risk factor for the long-term death of diabetic patients, and the definition of CA-AKI_A_ is more suitable for diabetic patients. This is helpful for clinical patients to identify CA-AKI patients who are highly related to long-term death among diabetic patients, so as to give more kidney protection measures to improve their prognosis.

## Data Availability

The datasets generated and/or analysed during the current study are not publicly available due data privacy regulation by Guangdong Provincial People's Hospital, but are available from the corresponding author on reasonable request.

## Abbreviations

CA-AKI: Contrast-associated acute kidney injury
CAG: Coronary angiography
PARs: Population attributable risks
HS-CRP: High-sensitivity C-reactive protein
AMI: Acute myocardial infarction
AKIN: Acute Kidney Injury Network
STEMI: ST-segment elevation myocardial infarction
IABP: Intra-aortic balloon pump
CHF: Congestive heart failure
NYHA: New York Heart Association
GFR: Glomerular filtration rate
ACEI: Angiotensin-converting enzymes inhibitor
ARB: Angiotensin receptor blocker
PCI: Percutaneous coronary intervention
SCr: Serum creatinine
